# Prevalence and associated risk factors of asymptomatic malaria and anaemia among school-aged children in Dara Mallo and Uba Debretsehay districts: results from baseline cluster randomized trial

**DOI:** 10.1186/s12936-021-03937-2

**Published:** 2021-10-13

**Authors:** Zerihun Zerdo, Hilde Bastiaens, Sibyl Anthierens, Fekadu Massebo, Matewos Masne, Gelila Biresaw, Misgun Shewangizaw, Abayneh Tunje, Yilma Chisha, Tsegaye Yohannes, Jean-Pierre Van Geertruyden

**Affiliations:** 1 Department of Medical Laboratory Science, College of Medicine and Health Sciences, Arba Minch University, Arba Minch, Ethiopia; 2 Global Health Institute, Antwerp University, Antwerp, Belgium; 3 Department of Family Medicine and Population Health, Antwerp University, Antwerp, Belgium; 4 Department of Biology, College of Natural Sciences, Arba Minch University, Arba Minch, Ethiopia; 5 Department of Public Health, College of Medicine and Health Sciences, Arba Minch University, Arba Minch, Ethiopia

**Keywords:** Prevalence of malaria, Altitude-adjusted haemoglobin, Predictors, School-aged children, Southern Ethiopia

## Abstract

**Background:**

Despite the growing evidence that malaria and anaemia are two interlinked health problems of school-aged children (SAC) in developing countries, there is scarce information about malaria among SAC in Ethiopia. Moreover, anaemia-related studies were more concentrated in easily accessible areas. This study aimed to assess the prevalence of malaria and anaemia and corresponding risk factors among SAC in Dara Mallo and Uba Debretshay districts, in hard to reach areas, so as to inform appropriate integrated interventions for both diseases.

**Methods:**

This study was part of baseline data collected for a cluster-randomized trial registered in Pan African Clinical Trials Registry (PACTR202001837195738). Data were collected from 2167 SAC and their households through face-to-face interview; malaria was diagnosed by using rapid diagnostic test (RDT); haemoglobin concentration was determined using hemoCue hb 301 and adjusted for altitude to determine anaemic status; helminth infections were determined by using kato-katz, and anthropometric measurements were made to determine nutritional status of children. Generalized mixed effects logistic regression model was used to assess the association between predictor variables and malaria and anaemia using school as a random variable.

**Results:**

The overall prevalence of malaria was 1.62% (95% CI 1.15–2.27%) (35/2167). Of the 35 children positive for malaria, 20 (57.14%), 3 (8.57%) and 12 (34.29%) were due to *Plasmodium falciparum*, *Plasmodium vivax* and mixed infections of *P. falciparum* and *P. vivax,* respectively. Malaria was significantly lower among children from literate household head (Adjusted OR  =  0.38; 95% CI 0.15–0.95) and residence house located at an altitude range above 1100 masl (AOR  =  0.40; 95% CI 0.17–0.94). The prevalence of anaemia was 22.00% (95% CI 20.3–23.8%) (477/2167) and was significantly reduced by eating legumes, nuts or seed group of food in their 24-h dietary diversity recall (AOR  =  0.64; 95% CI 0.41–0.99).

**Conclusions:**

The prevalence of malaria was low and unevenly distributed per school while the overall prevalence of anaemia was moderate. It is important to implement integrated interventions targeting both malaria and anaemia, with special emphasis given to children from illiterate households and living at an altitude below 1100 masl. The micronutrient content of locally grown legumes should be further investigated to recommend specific interventions to overcome anaemia.

## Background

Malaria is an infectious disease caused by *Plasmodium* species of protozoan parasites. There are five known *Plasmodium* parasites causing malaria in humans. *Plasmodium falciparum* and *Plasmodium vivax* are the two dominant species causing malaria in Ethiopia [1, 2]. Due to intensified malaria prevention measures, malaria substantially declined between 2010 and 2015 globally, but this decline was halted between 2015 and 2017. Thus, malaria remains a public health concern and a major cause of death and disease among infectious diseases in tropical and economically deprived areas [1–3]. According to the World Health Organization (WHO) Report 2020, there were an estimated 229 million malaria cases and 4,09,000 deaths globally in the year 2019. About 94% of those cases and more than 95% of deaths occurred in the WHO African region [4].

Nowadays, there is an age shift in the susceptibility to malaria. Malaria prevention measures targeting children under 5 years old leads to decreased exposure of them to *Plasmodium* species [5–7]. Less frequent exposure of children in their early life leads to delayed development of anti-malarial immunity. A study conducted in Malawi revealed that school-aged children (SAC) were as susceptible as under-five children to *Plasmodium* species [8]. As compared to pregnant women, SAC were at two-fold increased risk for malaria and 1% increase in malaria among pregnant mothers was equivalent to 4% increase in malaria among SAC in sub-Saharan Africa (SSA) [9]. The other explanation for high prevalence of malaria among SAC was the duration of persistent infection. Buchwald et al*.* [8] noted that the duration of persistent *Plasmodium* infection was 38% higher in SAC than children under 5 years old.

The prevalence of malaria and its determinants varies widely in the different contexts. Up to 73.9% of SAC in SSA were infected by *Plasmodium* species [10]. It was 47% in Equatorial Guinea [11], 10.8% in Malawi [12], 1.7–18.4% in Tanzania [13–15], 6.4–38.3% in Kenya [5, 16–18], 40% in South Sudan [19], and 73.9% in Côte d’Ivoire [20]. Malaria burden in this population segment was related to access and adherence to malaria prevention interventions, place of residence and certain socio-demographic factors [11, 17, 19–23]. Malaria was responsible for up to 50% death among SAC in SSA [10].

The other consequences of malaria are life-threatening anaemia and metabolic acidosis [10]. Anaemia is a physiologic condition in which the red blood cell mass is low to meet the physiological need (oxygen carrying capacity) of the body, or concentration of haemoglobin below the recommended threshold. The physiological need of an individual varies depending on age, gender, residence, altitude, smoking, and the different stages of pregnancy [24, 25]. *Plasmodium* infections cause anaemia through direct destruction of the red blood cells, clearance of infected and uninfected red blood cells by the spleen and impaired production of the red blood cells by the bone marrow [26, 27].

Anaemia was used as an important health indicator in the population. It is more common in economically deprived populations where malaria and other infectious diseases were highly prevalent. For each gross domestic product (GDP) increase in China, there was a 40% reduction in the prevalence of anaemia among SAC [28]. The major cause of anaemia in developing countries is iron deficiency. In addition, it is influenced by a child’s nutritional status, family income, gender, households owning poultry, household size, and residing in a rural area [11, 16, 17, 29–33]. Globally, about an estimated 305 million SAC were suffering from anaemia [26]. Articles reviewed indicate that the prevalence of anaemia among SAC ranged from 10.8% in Brazil [34] to 85.2% in Equatorial Guinea [11]. The estimated prevalence of anaemia was 73.3% among SAC in Haiti [29], 33.3% in Uganda [30] and 50.8% in Kenya [31].

Despite the dramatic reduction in malaria in the last two decades, malaria remains a major public health and economic problem in Ethiopia [4]. According to the national malaria elimination strategy in Ethiopia, about 52% of the total population are at risk of malaria. Ethiopia is classified into five distinct malaria transmission strata based on the annual parasite index (API). These are high malaria endemic (API  ≥  50), moderate endemic (API  ≥  10 and   <  50), low endemic (API  >  5 and  <  10), very low endemic (API  ≤  5 and  >  0) and malaria-free areas (API  =  0) [35]. Although there is growing evidence that SAC become vulnerable and serve as the main reservoir for malaria [5, 13, 21], there is no study primarily targeting SAC to assess the prevalence and associated risk factors. A stratified analysis of a recent whole population-targeted epidemiological study [36], national malaria indicator survey [37] and facility-based surveys [19] revealed that malaria was high in this population segment. Furthermore, studies of anaemia among SAC in Ethiopia were more focused in areas near to universities or research institutions, which are easily accessible [32, 38]. Studies primarily focusing on malaria and anaemia among SAC and the corresponding associated factors are of paramount importance for integrated intervention for this population segment. Thus, this study was aimed to assess the prevalence of malaria and anaemia and corresponding associated risk factors among SAC in Dara Mallo and Uba Debretsehay districts, hard-to-reach areas, by using data collected at baseline of a cluster-randomized controlled trial in October to December 2019 to assess the effect of malaria prevention education on malaria, anaemia and cognitive development of SAC.

## Methods

### Study setting

**Fig. 1:**
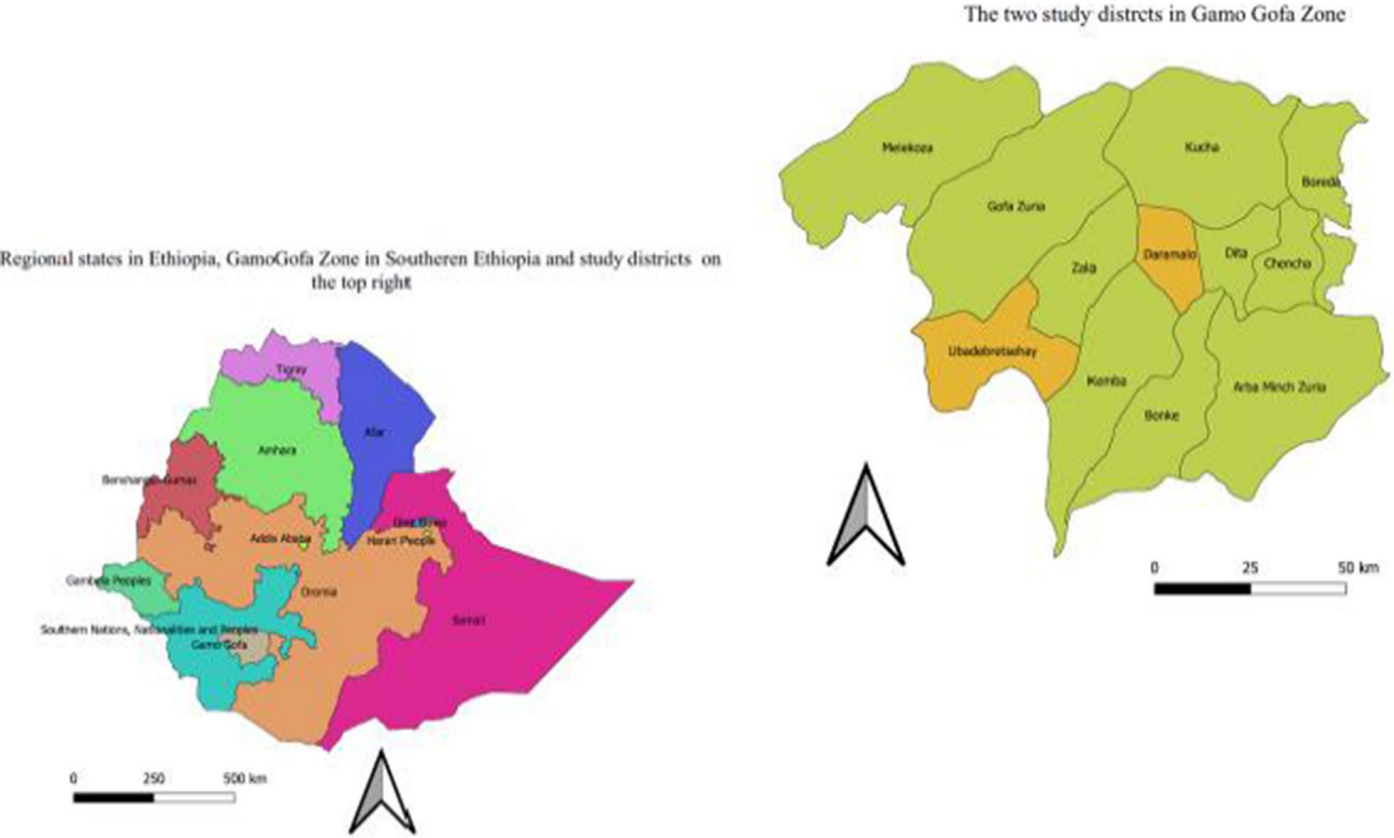
Location map of study districts in southern nations, nationalities and people’s region, Ethiopia

This study was conducted in Dara Mallo and Uba Debretshay districts in Gamo and Gofa Zones, respectively (Fig. 1). The population size, based on the 2007 national census [39], and the updated population in the study area was described previously [40]. Specifically, based on the 2007 national census, 1,50,145 people were living in the two districts, of whom 76,550 (51%) were male. According to the recent update made in 2020 by the respective districts, there were a total of 94,396 people in Uba Debretsehay district and 1,10,207 people in Dara Mallo district.

### Study design, sample size and sampling technique

This study was part of baseline data collected for a cluster-randomized trial evaluating the effect of malaria prevention education on malaria, anaemia and cognitive development of SAC. The trial was registered in Pan African Clinical Trials Registry (PACTR202001837195738). Study design, sample size estimation and sampling technique for the trial were described elsewhere [40].

### Method of data collection

The data collection process was carried out between October and December 2019. A pre-tested, structured questionnaire was uploaded to the Open Data Kit (ODK) data collection tool for observation and face-to-face interview with the mother of the child (caretaker). Socio-demographic factors, household assets, housing conditions, 24-h dietary diversity of SAC, sanitation and hygiene facilities, bed-net ownership and utilization-related factors were noted. The 24-h dietary diversity questionnaire was adapted from guidelines for measuring household and individual dietary diversity, developed by the Food and Agricultural Organization [41]. In addition, the coordinates of the household location were collected using global positioning system (GPS). Data collectors were trained on ethical aspects and on how to use the data collection tool. They were supervised daily for completeness and correctness of the data by supervisors from Arba Minch University.

### Diagnosis of malaria and haemoglobin determination

Except the face-to-face interview with mothers (caretakers) of the children, other components of the data were collected at the school compound by trained data collectors from Arba Minch University and health professionals from Dara Mallo and Uba Debretsehay districts, under close supervision of the principal investigators.

The finger prick blood specimen was collected to determine haemoglobin concentration by using HemoCue hb 301 [42] and *Plasmodium* infection status by using a rapid diagnostic test (RDT) (CareStart™ PF/PV(HRP2/pLDH)Ag Combo), which is 98 and 96% sensitive for *P. falciparum* and *P. vivax*, with equal specificity of 97.5% for both species. The auxiliary temperature of all children was taken by using a digital thermometer. Auxiliary body temperature above 37.5 °C and positive in RDT was considered as a clinical malaria case, otherwise the child was considered as an asymptomatic infection. *Plasmodium*-infected individual were treated according to the national malaria treatment guideline [43].

### Anthropometric measurements

Anthropometric measurements were taken according to WHO guidelines [44]. Children were weighed by using Seca digital scales placed on a hard and flat surface. Children wearing only lightweight clothing (excluding shoes, belts, socks, watches, and jackets) were weighed. Each child’s weight was measured to the nearest 0.1 kg by two individuals. If either measurements differed by more than 0.1 kg, a third measurement was made and the two closest measurements with a difference less than or equal to 0.1 kg were recorded.

When measuring the height of a child, he/she stood with his/her back against the board, his/her heels, buttocks, shoulders, and head touching a flat upright sliding head piece. The child’s legs were placed together with the knees and ankles brought together. Children were asked to take a deep breath, and the height measurement was taken at maximum inspiration. The head piece was brought down onto the uppermost point on the head and the height was recorded to the nearest 0.1 cm at the examiner’s eye level. Heights were measured by two data collectors and difference of 0.5 cm was tolerated. In cases when the difference between the two measurements was greater than 0.5 cm, a third measurement was taken and the nearest two measurements were recorded.

### Stool examination

After instructing children on how to collect stool specimen without contamination with soil or urine and any dirty material, a polyethylene screw-capped stool container was given to all children enrolled in the study in order to bring their stool specimen. Stool samples were collected at the school compound. The collected stool specimens were transported to Wacha and Beto health centres for laboratory diagnosis within the same day of collection. Stool smears were prepared following manufacturer’s instruction of Kato-Katz thick smear preparation. Prepared stools were left for 30 min after preparation and examined under bright field microscope within an hour. The template used for preparation of the smear was 41.7 mg [45]. The number of helminth eggs present in a whole smear was counted and written on a laboratory report form designed for the purpose.

### Data analysis

Data collected by using the ODK data collection tool were converted into a comma-separated value (CSV) files by using ODK briefcase. The average weight and height of a child was taken to represent their weight and height. The WHO AntrhroPlus software was used to calculate anthropometric indices such as body mass index (BMI)-for-age-z-score (BAZ), height-for-age-z-score (HAZ) and weight-for-age-z-score (WAZ). Children whose indices were less than − 2 were considered as underweight, stunted or wasted (for children age under or equal to 10 years), respectively for the previous indices. A child whose z-score was less than − 2 in any of the above indices was considered undernourished [46].

The haemoglobin concentration was adjusted for altitude based on WHO recommendations. Anaemia was defined based on WHO classification of haemoglobin (Hb) concentration: Hb  <  115 g/L for children aged 5–11 years; Hb  <  120 g/L for children aged 12–14 years [47]. The dietary diversity score (DDS) for the nine groups of foods, based on FAO recommendations, was computed [41].

Multiple factor analysis by using household assets, housing conditions, source of drinking water, agricultural land area, and the number of domestic animals was used to generate the wealth index of a household. The first dimension was classified into tertiles to classify household economic status into poor, medium and high.

Descriptive statistical analysis was performed; univariable and multivariable mixed effects logistic regression models were used to assess the association between malaria infection status and anaemia among SAC taking into account the cluster effect. The analysis was made using glmer function in lme4 R package. Odds ratio (OR) and corresponding 95% confidence interval (CI) were used to assess the strength of association between the outcome variables and predictor variables. Variables with P-value less than 0.25 in univariable analysis were potential candidates for multivariable mixed effect logistic regression. The fit of the model in predicting the outcome variables was checked by Akaike Information Criterion (AIC). Selection of variables for multivariable mixed effect logistic regression was made through backward stepwise variable selection method in which variable with the largest P value is removed from the model and checked for AIC. If removal of a variable from the model improves the AIC value, it is removed from the model, otherwise it is maintained in the model. For multivariable mixed effects models, P-value less than 0.05 were considered statistically significant.

## Results

A total of 2167 SAC participated in the study, which gave a response rate of 94.1% (2,167/2,304). Of these, 64.0% were from Uba Debretshay district, 85.0% were from rural areas, 50.1% were boys and the mean (SD) age of children was 8.75 (1.53) years. About 39.2, 28.3 and 32.5% of children were attending grades 1, 2 and 3 education, respectively. Household residence lies at an altitude between 754 and 1988 masl with mean (SD) altitude of 1192 m (145 m) masl (Table 1).

**Table 1:** Socio-demographic characteristics of participants per districts (Uba Debretsehay or Dara Mallo), Southern Ethiopia, 2019

Variable	Categories	N (%)	N (%)	
			Dara Mallo	Uba Debretsehay
Residence	Rural	1842 (85.00)	660 (84.51)	1182 (85.28)
	Semi-urban	180 (8.31)	109 (13.96)	71 (5.12)
	Urban	145 (6.69)	12 (1.54)	133 (9.60)
Gender of the household head	Male	2014 (92.94)	737 (94.37)	1277 (92.14)
	Female	153 (7.06)	44 (5.63)	109 (7.86)
Age of the household head	≤ 34	550 (23.38)	208 (26.63)	342 (24.68)
	35–49	1448 (66.82)	495 (63.38)	953 (68.76)
	≥ 50	169 (7.80)	78 (9.99)	91 (6.57)
Occupation of the household head	Farmer	1772 (81.77)	601 (76.97)	1171 (84.49)
	Civil servant	152 (7.01)	82 (10.50)	70 (5.05)
	Merchant	114 (5.26)	54 (6.91)	60 (4.33)
	Housewife	71 (3.28)	14 (1.79)	57 (4.11)
	Others	58 (2.68)	30 (3.84)	28 (2.02)
Educational status of household head	Illiterate	1193 (55.05)	271 (34.70)	922 (66.52)
	Literate	974 (44.95)	510 (65.30)	464 (33.48)
Occupation of the child mother/caretaker	Housewife	1794 (82.79)	623 (79.77)	1171 (84.49)
	Farmer	174 (8.03)	50 (6.40)	124 (8.95)
	Civil servant	80 (3.69)	49 (6.27)	31 (2.24)
	Merchant	87 (4.01)	41 (5.25)	46 (3.32)
	Others	32 (1.48)	18 (2.30)	14 (1.01)
Educational status of child mother	Illiterate	1569 (72.40)	450 (57.62)	1119 (80.74)
	Literate	598 (27.60)	331 (42.38)	267 (19.26)

## Malaria and its predictors

**Fig. 2:**
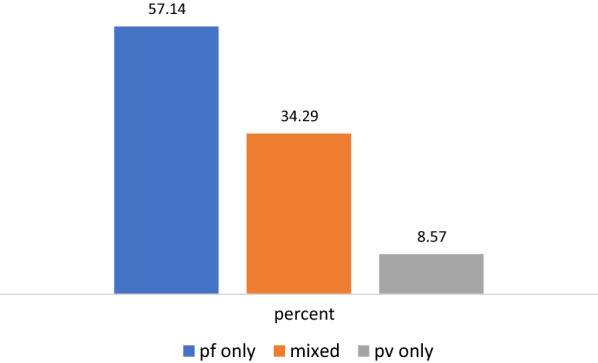
Distribution of *Plasmodium* species among infected children in Dara Mallo and Uba Debretsehay, southern Ethiopia, 2019

About 1.62% (35/2,167) of SAC were RDT-positive with 95% CI of 1.15–2.27%. Of the total, 35 SAC-positive for malaria 20, 3 and 12 were due to *P. falciparum*, *P. vivax* and mixed infection with *P. falciparum* and *P. vivax,* respectively (Fig. 2). The prevalence of malaria ranges from 0.0 to 17.1% in the schools involved in the study. None of the positive children had a temperature above 37.5 °C.

There was no *Plasmodium* infection among SAC residing at an altitude above 1500 masl. Household ownership of bed nets was not associated with malaria in univariable analysis (COR  =  0.76; 95% CI 0.28–2.06), but none of the children who slept under bed net the night before the survey was positive for malaria. The prevalence of malaria was 1.79% in rural areas while it was 0.62% in other areas although the difference was not statistically significant (COR  =  0.38; 95% CI 0.07–2.07). The prevalence of malaria was 1.75% in boys and 1.48% in girls with no statistically significant difference (COR  =  1.22; 95% CI 0.62–2.41). Literate educational status of household head (COR  =  0.34; 95% CI 0.15–0.80), malnourished children (COR  =  3.37; 95% CI 1.49–7.63), wasted children (COR  =  0.70; 95% CI 0.50–0.98), and being resident in a household located at an altitude above 1,109.89 masl (COR  =  0.40; 95% CI 0.17–0.94) significantly influenced malaria among SAC in univariable mixed effect logistic regression. However, after fitting the multivariable mixed effect logistic regression and taking care of collinearity between malnourishment and wasted children, only literate educational status of household head (AOR  =  0.38; 95% CI 0.15–0.95) and being resident in a household at an altitude above 1109.89 masl (AOR  =  0.40; 95% CI 0.17–0.94) remained statistically significant (Table 2).

**Table 2:** Univariable and multivariable analysis of factors affecting asymptomatic malaria among school-aged children in study districts

Characteristics	Categories	N (%)		COR (95% CI)	P value	AOR (95% CI)	P value
		−ve	+ ve				
Place of residence	Rural	1809 (98.21)	33 (1.79)	1	0.27		
	Not rural	323 (99.38)	2 (0.62)	0.38 (0.07–2.07)			
Age of the child in years	7–9	1568 (98.34)	26 (1.63)	1	0.56		
	10–14	564 (98.43)	9 (1.57)	0.83 (0.37–1.84)			
Gender of SAC	Male	1066 (98.52)	19 (1.75)	1.22 (0.62–2.41)	0.56		
	Female	1066 (98.25)	16 (1.48)	1			
Occupation of household head	Farmer	1739 (98.14)	33 (1.86)	1	0.10		
	Others	393 (99.49)	2 (0.51)	0.28 (0.06–1.26)			
Educational status of household head	Illiterate	1166 (97.74)	27 (2.26)	1	0.01	1	0.039
	Literate	966 (99.18)	8 (0.82)	0.34 (0.15–0.80)		0.38 (0.15–0.95)	
Occupational status of child mother/care giver	Housewife	1769 (98.61)	25 (1.39)	1	0.08		
	Others	363 (97.32)	10 (2.68)	2.13 (0.92–4.93)			
Education level of child’s mother/care giver	Illiterate	1543 (98.34)	26 (1.66)	1	0.84		
	Literate	589 (98.49)	9 (1.51)	1.09 (0.47–2.51)			
Is there pregnant mother in the household?	No	1871 (98.32)	32 (1.68)	1	0.73		
	Yes	261 (98.86)	3 (1.14)	0.81 (0.23–2.80)			
Is there under-five child in the household	No	726 (98.51)	11 (1.49)	1	0.88		
	Yes	1406 (98.32)	24 (1.68)	0.94 (0.45–2.00)			
Is there stagnant water around residence	No	1665 (98.55)	29 (1.45)	1	0.68		
	Yes	165 (96.53)	6 (3.49)	1.29 (0.39–4.34)			
Distance of the household from HF	≤ 5 km	1361 (98.84)	16 (1.16)	1	0.15		
	> 5 km	771 (97.59)	19 (2.41)	1.90 (0.79–4.60)			
Is there bed net in the household	No	1718 (98.28)	30 (1.72)	1	0.59		
	Yes	414 (98.81)	5 (1.19)	0.76 (0.28–2.06)			
Malnourished	No	1662 (98.64)	23 (1.36)	1	0.004	1	0.974
	Yes	188 (95.43)	9 (4.56)	3.37 (1.49–7.63)		1.0 (0.98–1.02)	
Wasted (WAZ)	No	1744 (98.48)	27 (1.52)	1	0.04		
	Yes	110 (95.65)	5 (4.35)	0.70 (0.50–0.98)			
Under weighted (BAZ)	No	2004 (98.43)	32 (1.57)	1	0.38		
	Yes	120 (97.56)	3 (2.44)	0.84 (0.57–1.24)			
Stunted (HAZ)	No	1919 (98.51)	29 (1.49)		0.32		
	Yes	205 (97.16)	6 (2.84)				
Wealth Index in tertiles	Low	704 (97.24)	20 (2.76)	1		1	
	Medium	714 (98.89)	8 (1.11)	0.44 (0.19–1.03)	0.05	0.42 (0.17–1.02)	0.056
	High	714 (99.03)	7 (0.97)	0.04 (0.16–1.01)	0.06	0.50 (0.18–1.38)	0.183
Altitude in MASL	< 1109.89	868 (97.42)	23 (2.58)	1	0.027	1	0.035
	> 1109.89	1263 (99.06)	12 (0.94)	0.38 (0.16–0.90)		0.40 (0.17–0.94)	

### Anaemia and its predictors

**Fig. 3:**
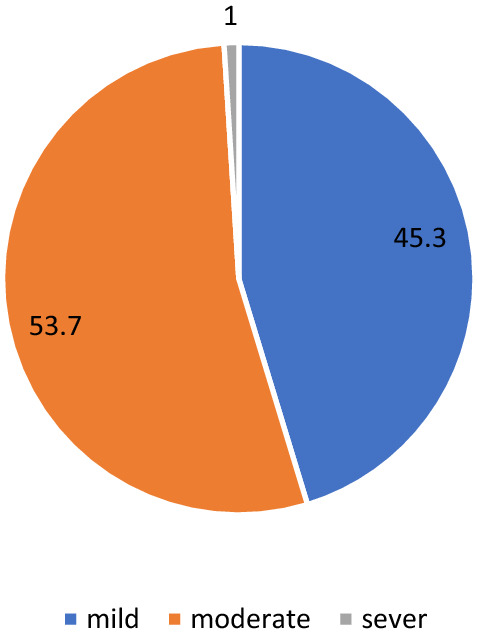
Level of anaemia in percentage among anaemic children in Dara Mallo and Uba Debretsehay, southern Ethiopia, 2019

The mean altitude adjusted Hb concentration was 12.43 (SD: 1.27) g/dL. The prevalence of anaemia was 22.0% (95% CI 20.3–23.8) (477/2167). Of the 477 anaemic SAC, 216, 256 and 5 were mild, moderate and severely anaemic, respectively. The proportion of mild, moderate and severe anaemia among anaemic children is depicted in Fig. 3.

No socio-demographic factors, soil-transmitted helminthes (STH) infections or infection by particular species of STHs, *Schistosoma mansoni* infection, infection by *Plasmodium* species and co-infection of *Plasmodium* species with STH or *S. mansoni* were statistically significantly associated with anaemia (Table 3).

**Table 3:** Univariable and multivariable analysis of factors affecting anaemia among school-aged children in study districts

Characteristics	Categories	N (%)		COR (95% CI)	P value	AOR (95% CI)	P value
		Not anaemic	Anaemic				
Place of residence	Rural	1430 (77.63)	412 (22.37)	1	0.246	1	0.201
	Not rural	260 (80.00)	65 (20.00)	1.00 (0.46–2.17)		1.23 (0.90–1.69)	
Age of the child in years	≤ 11	1575 (78.20)	439 (21.80)	1	0.437		
	12–14	115 (75.16)	38 (24.84)	1.18 (0.78–1.80)			
Gender of SAC	Female	856 (79.11)	226 (20.89)	1	0.116		
	Male	834 (76.87)	251 (23.13)	1.19 (0.96–1.48)			
Occupation of household head	Farmer	1369 (77.26)	403 (22.74)	1	0.144		
	Others	321 (81.27)	74 (18.73)	0.77 (0.54–1.09)			
Educational status of household head	Illiterate	914 (76.61)	279 (23.39)	1	0.611		0.485
	Literate	776 (79.67)	198 (20.33)	1.07(0.83–1.38)		0.92 (0.73–1.16)	
Occupational status of child mother/care giver	Housewife	1372 (76.48)	422 (23.52)	1	0.121		
	Others	318 (85.25)	55 (14.75)	0.75 (0.53–1.08)			
Educational status of child’s mother/care giver	Illiterate	1202 (76.61)	367 (23.39)	1	0.225		
	Literate	488 (81.61)	110 (18.39	0.84 (0.63–1.11)			
Is child positive for malaria	No	1666 (78.14)	466 (21.86)	1	0.721		
	Yes	24 (68.57)	11 (31.43)	1.15 (0.53–2.54)			
Is child infected by any STH?	No	1441 (79.06)	299 (20.76)	1	0.185	1	0.340
	Yes	549 (75.72)	176 (24.28)	1.18 (0.92–1.51)		1.12 (0.89–1.40)	
Is the child infected by hookworm?	No	1308 (78.18)	365 (21.82)	1	0.304		
	Yes	382 (77.33)	112 (22.67)	1.16 (0.88–1.53)			
Is the child infected by round worm?	No	1594 (78.18)	445 (21.82)	1	0.777		
	Yes	96 (76.19)	30 (23.81)	1.07 (0.67–1.69)			
Is the child infected by whipworm?	No	1509 (78.63)	410 (21.37)	1	0.996		
	Yes	181 (73.58)	65 (26.42)	1.00 (0.71–1.41)			
Is the child infected by *Schistosoma mansoni*?	No	1469 (77.85)	418 (22.15)	1	0.712		
	Yes	221 (79.50)	57 (20.50)	0.92 (0.59–1.43)			
STH-malaria co-infected	No	1681 (78.11)	471 (21.89)	1	0.756		
	Yes	9 (69.23)	4 (30.77)	1.22 (0.34–4.36)			
STH or SCH malaria co-infected	No	1679 (78.09)	471 (21.91)	1	0.974		
	Yes	11 (73.33)	4 (26.67)	1.02 (0.30–3.48)			
Wealth Index in tertiles	Low	545 (75.28)	179 (24.72)	1			
	Medium	559 (77.42)	163 (22.58)	1.03 (0.78–1.35)	0.841		
	High	586 (81.28)	135 (18.72)	0.89 (0.65–1.20)	0.434		

The mean DDS of children from 24-h dietary recall was 3.57 (SD: 1.74) from the nine food groups included in the analysis (Table 4). In univariable analysis, anaemia was statistically significantly associated with malnourishment (COR  =  1.66; 95% CI 1.15–2.42) and stunting (COR  =  0.92; 95% CI 0.85–0.99). However, after fitting the model in multivariable analysis, neither malnourishment nor stunting remained statistically significant while eating food groups in the legumes, nuts or seeds, or became associated with anaemia (AOR  =  0.64; 95% CI 0.41–0.99) (Table 4).

**Table 4:** Univariable and multivariable analysis of association between anaemia and nutritional status or dietary diversity

Characteristics	Categories	N (%)		COR (95% CI)	P value	AOR (95% CI)	P value
		Not anaemic	Anaemic				
Malnourished	No	1323 (78.52)	362 (21.48)	1	0.007	1	0.806
	Yes	144 (73.10)	53 (26.90)	1.66 (1.15–2.42)		1.00 (0.98–1.02)	
Wasted (WAZ)	No	1386 (78.26)	385 (21.74)	1	0.135		
	Yes	85 (73.91)	30 (26.09)	0.92 (0.82–1.03			
Under weighted(BAZ)	No	1585 (77.85)	451 (22.15)	1	0.883		
	Yes	98 (79.67)	25 (20.33)	1.01 (0.89–1.15)			
Stunted (HAZ)	No	1532 (78.64)	416 (21.36)	0.92 (0.85–0.99)	0.026	1	0.070
	Yes	151 (71.56)	60 (28.44)	1		1.07 (0.99–1.15)	
Starchy staples (cereals/white roots)	No	18 (85.71)	3 (14.29)		0.472		
	Yes	1672 (77.91)	474 (22.09)	1			
Dark green leafy vegetables	No	780 (76.70)	237 (23.30)	1.60 (0.44–5.77)	0.111		
	Yes	910 (79.13)	240 (20.87)	0.79 (0.58–1.06)			
Vitamin A-rich vegetables and fruits	No	598 (76.28)	186 (23.72)	1	0.754		
	Yes	1092 (78.96)	291 (21.04)	0.96 (0.74–1.25)			
Vegetables and fruits other than vitamin A-rich	No	780 (76.70)	237 (23.30)	1	0.111		
	Yes	910 (79.13)	240 (20.87)	0.79 (0.58–1.06)			
Meat, chicken and fish	No	1617 (78.00)	456 (22.00)	1	0.950		
	Yes	73 (77.66)	21 (22.34)	0.98 (0.51–1.88)			
Organ meat	No	1617 (78.16)	452 (21.84)	1	0.584		
	Yes	72 (74.23)	25 (25.77)				
Eggs	No	1526 (78.54)	417 (21.46)	1	0.464		
	Yes	164 (73.21)	60 (26.79)	1.10 (0.74–1.62)			
Legumes, nuts and seeds	No	1,538 (77.60)	444 (22.40)	1	056	1	0.045
	Yes	152 (82.16)	33 (17.84)	0.64 (0.41–1.01)		0.64 (0.41–0.99)	
Milk and dairy products	No	703 (81.08)	164 (18.92)	1	0.238		
	Yes	987 (75.92)	313 (24.08)	1.17 (0.90–1.50)			

## Discussion

There is growing evidence that SAC become highly vulnerable to malaria and associated morbidities. They are playing a major role in the transmission dynamics of malaria. The prevalence of malaria among SAC in Dara Mallo and Uba Debretsehay districts was 1.62% and it was affected by educational status of the household head and the household location above 1,109.89 msal. Mean altitude-adjusted Hb concentration and anaemia among SAC were 12.43 g/dL and 20.0%, respectively. Anaemia was low amongst children who ate vegetables, nuts and seeds in their 24-h self-reported dietary recall.

The point prevalence of malaria in the present study area was low. Since all infections were asymptomatic, such individuals went undiagnosed but contribute the major role in the transmission dynamics of malaria. A study conducted in Malawi demonstrated that asymptomatic malaria infections persist for long periods in this population. WHO recommended identification and treatment of all infections of malaria in its Global technical strategy (GTS) for malaria, 2016–2030. There was a high heterogeneity in the burden of malaria in schools participating in the study, which calls for well-tailored malaria prevention interventions where prevalence of malaria is high. High uneven spatial distribution of malaria was also demonstrated in the previous epidemiology study in Ethiopia [36].

The prevalence of malaria in the present study area was in agreement with the last national malaria indicator survey of Ethiopia, the recently stratified estimate made in Arba Minch Zuria district [36] and one similar study undertaken in Tanzania [15]. In contrast, the prevalence of malaria in the present study area was lower than findings in Tanzania [13, 14], Kenya [5, 16–18], South Sudan [48], Côte d’Ivoire [20], demonstrated in stratified data analysis in some studies [19, 37]. The low prevalence of malaria might be attributable to the difference in level of malaria endemicity or different interventions undertaken prior to the data collection period. There was high malaria transmission in the study area before the data collection period. In response to this, different levels of health systems in the country were involved to overcome the transmission of malaria in the area. In addition to regular malaria prevention interventions in Ethiopia, screening and treatment at community level was done to halt the high transmission of malaria. One of the consequences of malaria is anaemia since the parasites destroy red blood cells after completing the developmental life cycle [47].

Unlike the previous hypothesis that malaria was significantly associated with anaemia, this was not demonstrated in the present study. Failure to demonstrate significant association between malaria and anaemia in this study might be due to the low number of *Plasmodium*-infected children and subsequent loss of power to detect the difference of anaemia among *Plasmodium*-infected and uninfected children. The prevalence of anaemia was moderate among SAC in Dara mallo and Uba Debretsehay districts. It was lower than prevalence of anaemia in similar population groups in Jima town [49], Arba Minch Zuria district [50] and the national pooled estimate in Ethiopia [38], while it was higher than the estimate made in Durbete town [32]. This finding was higher than the estimate made in Brazil [34] while it was lower than the prevalence of anaemia among SAC in Haiti [29], Uganda [30] and Kenya [31]. The differences in the estimated prevalence of anaemia compared to estimates made in Ethiopia and other developing countries might be related to variation in level of socio-economic development [28], and low prevalence of malaria and lower intensity of parasitic infections such as STH and intestinal schistosomiasis which did not significantly affected anaemia in the current study. Unlike this study, parasitic infections among SAC was one of the predictors of anaemia in Arba Minch Zuria district [50] and northern Ethiopia [51]. This difference might be related to difference in the type of parasitic infections involved in the diagnosis.

Apart from parasitic infections [49, 52, 53], anaemia is influenced by nutritional status [47] of children, which was not revealed after taking care of potential confounders in the present study. It was similar to the study conducted in Arba Minch Zuria district as stunting was not associated with altitude-adjusted anaemia [50], but in contrast, a community-based survey conducted in Gonder town [53] and the systematic review and meta-analysis of articles originated from Ethiopia [38]. The insignificant association between nutritional status and anaemia might be due to high prevalence of non-nutritional anaemia. Among the dietary diversity that significantly reduced anaemia in SAC was the category of vegetables, nuts and seeds. The significant effect of this dietary category on anaemia may be related to high iron and other micronutrient content, important for growth and maturation of normal red blood cells. The significant influence of eating legumes was also demonstrated by a study conducted in eastern Ethiopia [54]. Unlike a study conducted in South Africa [55], the insignificant association of food groups obtained from animals sources could be related to such foods being taken very rarely or inadequately in quantity, mainly in rural parts of the study area. The significant association between legumes, nuts and seeds in this study was corroborated by a cluster-randomized trial, where taking bio-fortified or not-fortified beans improved the Hb concentration among SAC in both the control and intervention arms [56]. The beans grown in rural parts were considered “meat of the poor” [57], to satisfy the nutritional demand of people who were at high risk of anaemia.

There were certain limitations associated with this study. The first was due to the design in which both outcome and predictor variables were assessed at the same time. Such studies were not strong in generating causal evidence. Secondly, the low power of the study to detect significant difference of malaria in its predictor variables and malaria as predictor for anaemia. The third limitation was related to the biomass of malaria-positive individuals. The number of *Plasmodium* parasites per μl of blood or the percentage of *Plasmodium*-infected red blood cells was not calculated. The fourth limitation is due to recruiting only school-enrolled children into the study as the situation may be different for children not enrolled to a school. The strength of this study is the area of the study being conducted in hard-to-reach areas, not previously explored.

## Conclusions

The overall prevalence of malaria among SAC in Dara Mallo and Uba Debretsehay districts was low, but there was uneven distribution of malaria in the schools. Malaria was more common among children from household heads who were illiterate and where the residence was located at an altitude range below 1100 masl. This calls for targeted intervention in schools where the prevalence of malaria is high with due emphasis given to schools at an altitude below 1100 masl and households with illiterate household heads. Anaemia was moderate in the area, being eligible for interventions to improve the haemoglobin concentration of SAC. Anaemia was negatively affected by 24-h dietary recall of eating legumes, nuts and seeds. Studies evaluating the micronutrient content of these foods and their daily intake for nutritional adequacy shall be studied.

## Data Availability

The datasets used and/or analysed during the current study will be available from the corresponding author on reasonable request.

## Abbreviations

AIC: Akaike information criterion
AOR: Adjusted odds ratio
API: Annual parasite index
BAZ: BMI-for-age-z-score
BMI: Body mass index
CI: Confidence interval
COR: Crude odds ratio
DDS: Dietary diversity score
DHS: Demographic and Health Surveys
GDP: Gross domestic product
GPS: Global positioning system
GTS: Global technical strategy
HAZ: Height-for-age-z-score
hb: Hemoglobin concentration
IRB: Institutional research ethics review Board
MASL: Meters above sea level
ODK: Open data kit
OR: Odds ratio
PACTR: Pan African Clinical Trials Registry
Pf: *P. falciparum*
Pv: *P. vivax*
QGIS: Quantum geographical information system
RDT: Rapid diagnostic test (RDT)
SAC: School-aged children
SD: Standard deviation
SSA: Sub-Saharan Africa
STH: Soil-transmitted helminths
WAZ: Weight-for-age-z-score
WHO: World Health Organization
